# Patient's behavior of selection physician in online health communities: Based on an Elaboration likelihood model

**DOI:** 10.3389/fpubh.2022.986933

**Published:** 2022-10-03

**Authors:** Min Qin, Wei Zhu, Changmeng You, Shuqin Li, Shanshan Qiu

**Affiliations:** ^1^Research Center of Management Science and Engineering, Jiangxi Normal University, Nanchang, China; ^2^School of Software, Jiangxi Normal University, Nanchang, China

**Keywords:** online health communities, Elaboration likelihood model, trust source theory, online word of mouth, patient selection behavior

## Abstract

**Background:**

With the rapid development of “Internet + medicine” and the impact of the COVID-19 epidemic, online health communities have become an important way for patients to seek medical treatment. However, the mistrust between physicians and patients in online health communities has long existed and continues to impact the decision-making behavior of patients. The purpose of this article is to explore the influencing factors of patient decision-making in online health communities by identifying the relationship between physicians' online information and patients' selection behavior.

**Methods:**

In this study, we selected China's Good Doctor (www.haodf.com) as the source of data, scrapped 10,446 physician data from December 2020 to June 2021 to construct a logit model of online patients' selection behavior, and used regression analysis to test the hypotheses.

**Results:**

The number of types of services, number of scientific articles, and avatar in physicians' personal information all has a positive effect on patients' selection behavior, while the title and personal introduction hurt patients' selection behavior. Online word-of-mouth positively affected patients' selection behavior and disease risk had a moderating effect.

**Conclusion:**

Focusing on physician-presented information, this article organically combines the Elaboration likelihood model with trust source theory and online word-of-mouth from the perspective of the trusted party–physician, providing new ideas for the study of factors influencing patients' selection behavior in online health communities. The findings provide useful insights for patients, physicians, and community managers about the relationship between physician information and patients' selection behavior.

## Introduction

The development of Internet technologies has promoted the maturation of online community models. Online health communities (OHCs) are rapidly gaining popularity as a type of healthcare community (1), becoming a quintessential model of “Internet + healthcare,” bringing together medical and health-related information (2). A variety of online healthcare services have emerged along with this, including an online search for health information, online consultation, appointment registration, and other services (3), as an important supplement to the offline medical system, online health communities provide a new channel for physician–patient communication and effectively alleviate the problem of difficult and expensive access to medical care (4). Online medical care breaks through the boundaries of geography and time, enabling patients to obtain health-related information at any time, make full use of medical and health resources, simplify the process of patient access, and improve the efficiency and quality of patient access. According to CNNIC, the size of online consultation users in China reached 298 million by December 2021 due to the COVID-19 epidemic (5). The rapid emergence of internationally renowned Internet healthcare platforms such as Teladoc, Good Doctor, Doctor on Demand, and PatientsLikeMe (6, 7) around the world has broken the limitations of space and time, saved costs, and treatment expenses, and protected the privacy of users. Therefore, more and more users are developing the habit of seeking health consultations online (8). During the COVID-19 epidemic, the number of consultations by the Good Doctor medical platform increased eight times, providing nearly 49 Million Online Consultations in 2021 (9), and online medical consultation will become the new trend in medical treatment.

Online health communities provide people with an important online place where they can search for health information, consult with doctors online, and exchange disease-related treatment experiences (10), mainly containing personal information of physicians and online word-of-mouth generated by patients (11). Online health community user behavior mainly includes knowledge sharing behavior, online health information searching behavior, selecting physician consultation and physician–patient interaction, online health management, etc. (12). Generally, after entering an online health community, patients need to select a suitable physician for consultation from among many physicians who provide online consultation services according to their conditions. However, the selection of different physicians and the online information of physicians can cause a certain information load (13), and the nature of healthcare services as a trusted commodity makes a serious information asymmetry between physicians and patients (14), which affects the selection of patients and increases the risk of their use (15). As service providers in the physician–patient relationship, physicians are an important guarantee of the online healthcare process and have a profound impact on the online physician–patient relationship, while medical health service is a high-trust demanding service, and patients may face the dilemma of selecting a quality physician from among many (16). How to make a good judgment and select a satisfactory physician based on the personal information and word-of-mouth information displayed by the physician has not only troubled patients but also aroused the interest of scholars.

A large number of previous studies have explored the factors influencing the selection of physicians. Early studies focused on offline physician–patient interactions, which occurred in brick-and-mortar hospitals and clinics (17), where physicians were always dominant in the traditional asymmetrical physician–patient relationship due to their medical expertise and the lack of freedom to select physicians as patients were required to obey hospital arrangements when seeking care (18). The influencing factors are mostly physician title, hospital rank, city, service attitude, and external word-of-mouth (10). The emergence of OHC has changed the way of physician–patient communication and service, and the existing studies on medical selection mainly focus on patient-generated word-of-mouth information (19), from the number of online reviews (20), positive and negative evaluations (21), online ratings (12), etc., and mostly use cross-sectional data such as questionnaires or experiments (11). As the provider of online physician–patient trust, physicians are the leaders of online medical services, and physicians' personal information is a direct indication of their trustworthiness. Based on this, this article constructs a multiple linear regression model from the information of physicians' OHC, with the Elaboration Likelihood Model theory as the structure, where the central route combines trust source theory and the peripheral route combines online word-of-mouth theory, to explore the influencing factors of patients' selection behavior and the moderating effect of disease risk on online word-of-mouth.

## Literature review

### OHC and patients' selection behavior

Online health communities are Internet platforms that provide health-related activities such as information search, disease consultation, experience sharing, and providing mutual encouragement for groups of people with the same disease or the same characteristic attributes (22). OHC can be divided into three categories according to the main participants in the online health community: ① Online patient communities: These communities are primarily composed of patient participation and exist in the form of forums and postings where patients can communicate and share their conditions, and provide a variety of information and emotional support (23). ② Online physicians community: The main participants are physicians, health industry personnel, and researchers in related fields. Physicians can conduct various exchange activities on the platform to learn from others' treatment experiences, expand their treatment horizons and improve their medical skills, as represented by the Lilac garden medical website (24). ③ Online physician–patient community: A media platform used for communication by physicians and patients, and based on which various professional medical services such as disease consultation and online consultation are provided. Representative websites include Good Doctor, 39Health.com, MedHelp, etc. (8, 25).

This article focuses on patient selection behavior in physician–patient communities. Physician behaviors in OHC mainly include: regularly updating personal information and consultation information, publishing articles, and responding to patient inquiries (8). Patient behaviors mainly include browsing and searching for information, selecting physicians, consulting physicians, and online evaluation (16). Current research on patient behavior in OHC focuses on online health information-seeking behavior (26), knowledge sharing behavior (27), online health information-seeking behavior (28), physician–patient interaction, healthcare service research (29), online health management (30), and other aspects. Much of the research on patient selection behavior has been done from a trusted perspective. Trust is one of the important components of the physician–patient relationship (31). While healthcare is a high-trust service, patients may face difficulties in judging the quality of services and making selections about the treatment options available to them (32). Quan et al. (10) confirmed that factors such as physicians' offline reputation and physicians' titles have an impact on patients' medical selection industry based on consumer trust theory. Gong et al. (11) explored, based on the trust theory, that the personal qualities of physicians and online reputation have an impact on patients' selection of physicians. In addition, physician reputation is often used by scholars to explore the impact on patient selection. Han et al. (33) explored the impact of online reviews (positive and negative reviews) on consumers' selection of physicians through a scenario-based experiment. Yuqi et al. (34) used data from third-party platforms for medical services to confirm the impact of online reputation on patients' selection of medical care. Shan et al. (31) used online trust theory to conduct an eye-tracking experiment and found that patient-produced word-of-mouth information influenced patients' selection of physicians through cognitive perception trust and affective trust. However, most of these studies related to trust in online health have been conducted from the trusting party–patient perspective, directly exploring the subjectively perceived trust of the giver. For patients, the information presented by physicians is a signal that their attributes can characterize their trustworthiness to a certain extent, while the online word-of-mouth of physicians is also a signaling mechanism that indirectly conveys trustworthiness. Therefore, it is necessary to explore how the personal information and online word-of-mouth of the trusted party–physician influence the decision-making of the trusting party–patient through the signaling mechanism of trust.

### Elaboration likelihood model

The elaboration likelihood model (ELM) is a classical theory of information influence route in the field of psychology proposed by Cacioppo and Petty (35). It states that the persuasion process consists of two routes that lead to attitude change, the central route, and the peripheral route with the difference lying in the level of the individual's likelihood of elaboration processing of the exposed information. The central route is related to the content of the information, and individuals who process information through the central route tend to think deeply about the relevant arguments in the information and make analyses and judgments (36). Individuals who process information through the peripheral route pay less attention to the quality of the information itself and rely heavily on the credibility of the source and the environmental characteristics of the information to judge the credibility of the target (36, 37). The impact of routes can vary as each user has a different level of knowledge base, ability, and motivation to engage in information processing (38). The main variables of the central route include information quality or content quality and the main variables of the peripheral route include source credibility and electronic word-of-mouth (39).

Elaboration likelihood model has been widely used in social media and e-commerce to verify users' attitudes or trustworthiness judgments on online reviews (40), social media messages (41), and second-hand information posting contents (42). Some existing studies explore applications in online communities, e.g., Shi et al. (43) explore factors influencing users' information dissemination behavior on online social networking sites based on the elaborate likelihood model. Bao and Wang (44) extend the understanding of the consumer information adoption process in brand microblogs from central and peripheral routes based on ELM. Wang et al. (45) analyzed the factors influencing the probability of increasing the likelihood of an idea being pre-selected or reviewed based on an ELM survey of Xiaomi MIUI community data. However, the ELM has rarely been applied in research on the patient selection of medical treatment in OHC. Only Xianye et al. (46) explored the effect of physician response information and online word-of-mouth on patients' intention to seek care through ELM in OHC. In the online healthcare field, misidentification of physician information and access choices can pose significant access risks; therefore, it is necessary to further explore the impact of different physician information on patients' medical selection through ELM models.

### Trust source theory

Mayer et al. (47) define trust as the willingness of the trusting party to demonstrate its vulnerability to the trusted party, regardless of its control and regulatory capacity, based on the expectation that the trusted party will behave beneficially. Sako and Helper (48) have argued that an explicit strategy for nurturing and sustaining trust can only be feasible if the determinants of trust are identified. The issue of determinants or drivers of trust is also known as the “trust source.” Mayer et al. (47) constructed a trust model from a dynamic perspective, identifying the components of trustworthiness of trust sources as benevolent, ability, and integrity trust. With the emergence of the Internet in recent years, the trust source theory has attracted more and more attention. An example is Wu et al. (49) who studied the impact of online responses from landlords on listing sales based on the trust source theory. Bansal et al. (50) argue that trust can be considered as a multidimensional construct of specific beliefs, and he finds that consumers' shopping intentions on Amazon.com are influenced by the reliability of the website's capabilities. Lu and Wu (16) found that trust in the ability of Taobao merchants positively stimulated consumers' purchase intentions in terms of benevolence, ability, and integrity.

In the online healthcare field, one of the important factors for the success of online health services is physician–patient trust. Existing scholars have conducted studies on physician–patient trust, such as (51), who found that the trustworthiness of websites, hospitals, and physicians, as well as perceived benefits and perceived risks, have significant effects on online patient trust. Yi et al. (52) found that argument quality, source expertise, perceived information quality, and perceived risk significantly affect users' trust in online health information. Shan et al. (31) found that the frequency of physician responses and the number of services willing to be opened positively influenced patients' selection behavior based on a trust source credibility model. As a provider of services in an online health community, the physician's personal information is an important trust source. Healthcare is a high-trust demanding service and patients may face difficulties in not being able to determine the quality of the service and make selections about the treatment options available to them (16). Trust as one of the important guarantees of relational exchange has a profound impact on the online physician–patient relationship; therefore, it is necessary to study the patient's selection behavior in online health communities from the perspective of patient trust. This article classifies patient trust in terms of information presentation. Benevolent, ability, and integrity trust in the information sources were used to represent patients' trust in different dimensions of physician information.

### *Online* word-of-mouth

Word-of-mouth is a verbal exchange of information about products, brands, services, vendors, etc. between consumers without the purpose of commercial promotion (53). It plays an important role in influencing consumer selection and brand formation (54). Gelb and Johnson (55) were the first to introduce the concept of “online word-of-mouth (OWOM),” which they considered a form of word-of-mouth communication that also includes the communication and exchange of information through the Internet. Consumers share their shopping experiences and reviews online, especially in common areas of interest such as movies, books, and restaurants (25). Compared with traditional word-of-mouth, OWOM has the characteristics of two-way interaction and low cost, which can break through the original spatial and temporal boundaries of communication (56). In recent years, several Internet healthcare third-party platforms have also introduced online reviews of physicians, creating an OWOM in healthcare (19).

A growing body of literature in recent years has begun to focus on the impact of OWOM on patients' selection of medical treatment. The impact of OWOM on patients' probability of booking a physician appointment is mainly discussed (12, 21), with less attention paid to the impact of OWOM on the selection of online healthcare services. For example, Bensnes and Huitfeldt (20) studied the effect of online ratings of Norwegian primary care physicians on the volume of their appointment (offline) services and found that physicians with high ratings served a significantly higher number of patients than those with low ratings. Xu et al. (12) constructed a BLP-type model to portray the heterogeneity of patients' selection of physicians in a US online physician appointment platform by extracting information from the reviews and found that higher ratings significantly increased the probability of a physician being appointed. Shukla et al. (19), using data from an Indian online physician appointment platform, found that the introduction of OWOM had a “cannibalization” effect, whereby the demand for physicians with high ratings increased significantly, cannibalizing the services of physicians without ratings. This article enriches the evidence related to Internet healthcare services by using data from a large Chinese online healthcare platform. In particular, this platform integrates online physician reviews and Internet medical services on a single website. This allows this article to simultaneously examine the impact of online physician information and OWOM on patient selection behavior.

## Research hypotheses and research model

### Physician information and central route

Information content and information quality are the main variables of the central route (39), involving the persuasive strength of the evidence embedded in the information (57). Studies have shown that in the online healthcare field, physician information is one of the most important considerations for patients when selecting healthcare services (31), with the content of information influencing the recipient's perceived usefulness of the information, which in turn influences behavior (57, 58). This article classifies physician–patient trust into benevolent trust, ability trust, and integrity trust based on Mayer et al. (47) three-factor division of trustworthiness of trust sources.

Benevolent trust is the willingness of the trusted party to do something beneficial to the trusting party out of altruistic motives. When the trusted party is perceived to be benevolent, the trusting party increases trust (11). Singh and Sirdeshmukh (59) point out that the more services an agent provide to a principal and the harder they serve, the stronger their benevolent trust is represented. In OHC, physicians, who may not be able to respond to patients' inquiries on the platform promptly due to busy schedules such as offline diagnosis or surgery may have a certain negative impression from the patients due to concerns about their unavailability, which may affect selection. From the patient's perspective, the more frequently the physician logs in and uses the online platform, the more viscous the physician is to the platform and the more importance they attach to patient matters, which enables the patient to perceive stronger trust in the physician's benevolence. In addition, a physician's benevolence is reflected in the degree to which they are accountable to patients, and a physician doing their job is an important indicator of good faith trust. Professionally responsible physicians create more opportunities to communicate and engage with patients by opening more types of health services and increasing the number of hours they are online. For physicians who open more types of services, the patient's selection of consultation services will not be limited to the physician's online consultation only, which can meet the patient's treatment needs. Therefore, we propose the following hypothesis.

***H1a:*** *The more recent the physician was last online the more patients selected that physician*.
***H1b:*** *The number of services opened by the physician positively influences the patient's selection behavior*.

Ability trust refers to the skills or talents that enable the trusted party to influence a particular area. A trusted party is only worthy of being trusted if they are highly competent in some specific area (11). Patients use medical skill level and professional competence to make selection decisions (51). The medical title level of physicians is generally formed by the comprehensive certification of physicians' academic level, working years, and professional qualification level (60). Physicians often also undertake scientific research tasks in universities, and their academic titles are the titles granted by their universities to show their scientific and academic abilities in their professional fields. Therefore, the physician title in this article is composed of both medical and academic titles. In addition, the number of physician's articles, both original and shared, which are published on the platform by the physician to help patients and make them aware of disease help patients judge the professional level of physicians. Therefore, we propose the following hypothesis.

***H2a:*** *Physician's title positively influences patients' selection behavior*.
***H2b:*** *The number of physician articles positively influences patients' selection behavior*.

Integrity trust refers to the willingness of the trusted party to reveal their true information without concealment and proactively promote trust by reducing information asymmetry and information risk present in online interactions (11). In OHC, where users perceive a great deal of information risk due to the virtual nature of the web, disclosing true information is one of the ways to enhance trust. While online consultation is about patients' personal life and health, patients are more inclined to trust physicians who can honestly present more personal information. Physicians can select whether to use their real avatars and fill in their biographies and professional fields with the level of detail of their biographies and professional fields when registering on the platform varies from person to person. In this article, the above three indicators are used as a measure of the degree of physician disclosure, i.e., an indication of integrity and trust in physicians. Therefore, we propose the following hypothesis.

***H3a:*** *Physicians use of avatars positively influences patients' selection behavior*.
***H3b:*** *Physician's introduction positively influences patients' selection behavior*.
***H3c:*** *Physician's professional field positively influences patients' selection behavior*.

In the field of information systems, trust is often classified into two different types, initial trust and continuous trust depending on the stage of formation (61). As the frequency of physician–patient interactions increases and relationship development progresses, the intensity of trust changes. Manski (62) argues that users in social networks are a group in nature and individual behavior is not only influenced by their own psychological and physical characteristics but also by the behavior of other users. In OHC, it is influenced by similar groups of users, who use a decision-making behavior based on information about similar patients who have previously selected that physician. The number of patient consultations after offline diagnosis measures the patient's continued trust in the physician. A higher number of patient consultations after offline diagnosis represents more patients who approve of the physician's services, which will increase the level of trust in the physician by other potential patients. Therefore, we propose the following hypothesis.

***H4:*** *The number of patients' consultations after a physician's offline diagnosis positively influence patients' selection behavior*.

### OWOM and peripheral route

Peripheral routes involve meta-information about the information (e.g., the source of the information) that is not contained in the information and is preferred by information receivers to aid in decision-making when they lack the ability and motivation to process the information (36). OWOM information is usually used as a peripheral route in ELM models (57, 63). In the e-commerce environment, OWOM is an effective signal of product quality and to some extent affects product sales (64). He et al. (65) found that OWOM influences final purchase behavior by enhancing consumers' trust in merchants. See-To and Ho (66) found that OWOM increases consumers' trust in merchants by trusting subjects' perception of trust in third-party evaluations influencing purchase intentions. Gottschalk and Mafae (67) argued that OWOM has a significant impact on users' decisions. In OHC, OWOM for physicians is divided into patient-generated and platform-generated (19), such as the number of patients' votes, thank-you letters, virtual gifts generated by patients, and the comprehensive recommendation hotness generated by the platform. The number of patients' votes, thank-you letters, and virtual gifts is the quantity of physicians' OWOM, which characterizes physicians' ability and service from the quantitative perspective, while the comprehensive recommendation hotness is the quality of physicians' OWOM, which characterizes the recognition and affirmation of physicians from the qualitative perspective. Comprehensive recommendation hotness is a platform that indicates to some extent a physician's contribution to the community based on their past performance, such as the length of online service and satisfaction with efficacy. Patients can perceive trust through OWOM, which can facilitate decision-making. Therefore, we propose the following hypothesis.

***H5a:*** *The number of patient's votes for physicians positively influences patients' selection behavior*.
***H5b:*** *The number of thank-you letters from physicians positively influences patients' selection behavior*.
***H5c:*** *The number of virtual gifts of physicians positively influences patients' selection behavior*.
***H5d:*** *The comprehensive recommendation hotness of physicians positively influences patients' selection behavior*.

### Moderating effect of disease risk

In the online shopping environment, consumer purchase decision behavior is differentially influenced by the moderating effects of product type and consumer characteristics (68). In the online health field, the factors influencing patients' healthcare selection behavior can also be influenced by the type of disease and the psychological characteristics of the patient. For patients suffering from different diseases, their perceived needs and involvement vary (69). Disease risk measures the severity of the consequences of a particular type of disease (70). The risk of disease is related to physical factors (i.e., health status) and physiological factors (i.e., distress, anxiety) (71). The more severe the physical and physiological consequences, the higher the risk of disease. Patients with high-risk disorders may have poorer health status than those at lower risk of developing the disease. Patients at high risk will be more worried and will have a greater desire to find higher quality physicians (16). In addition, patients with high-risk diseases require higher quality services than patients with low-risk diseases (60). Because of its association with mortality, patients with high-risk diseases may be more motivated to make more cognitive efforts to obtain a better physician. Compared to patients with low disease risk, patients with high-risk diseases have a greater cognitive demand for fact-based information presented by physicians, and cognitive demand affects the degree to which users process information (72). Patients with high cognitive demand may be more motivated to exert more cognitive effort to process fact-based information related to the physician themselves, i.e., have a high level of involvement (73). Patients with different diseases attach different importance to the physician's online reputation. When the patient's disease is serious, the patient will consider the physician's serviceability more comprehensively, and negative reviews will have more influence than positive reviews at this time with the patient shunning a physician with negative reviews. When the disease is mild, the patient believes that it will be cured soon and only wants to receive treatment as soon as possible. Therefore, we propose the following hypothesis.

***H6a:*** *Disease risk significantly moderates the relationship between the number of patients' votes and patients' selection behavior*.
***H6b:*** *Disease risk significantly moderates the relationship between the number of thank-you letters and patients' selection behavior*.
***H6c:*** *Disease risk significantly moderates the relationship between the number of virtual gifts and patients' selection behavior*.
***H6d:*** *Disease risk significantly moderates the relationship between comprehensive recommendation hotness and patients' selection behavior*.

**Figure 1:**
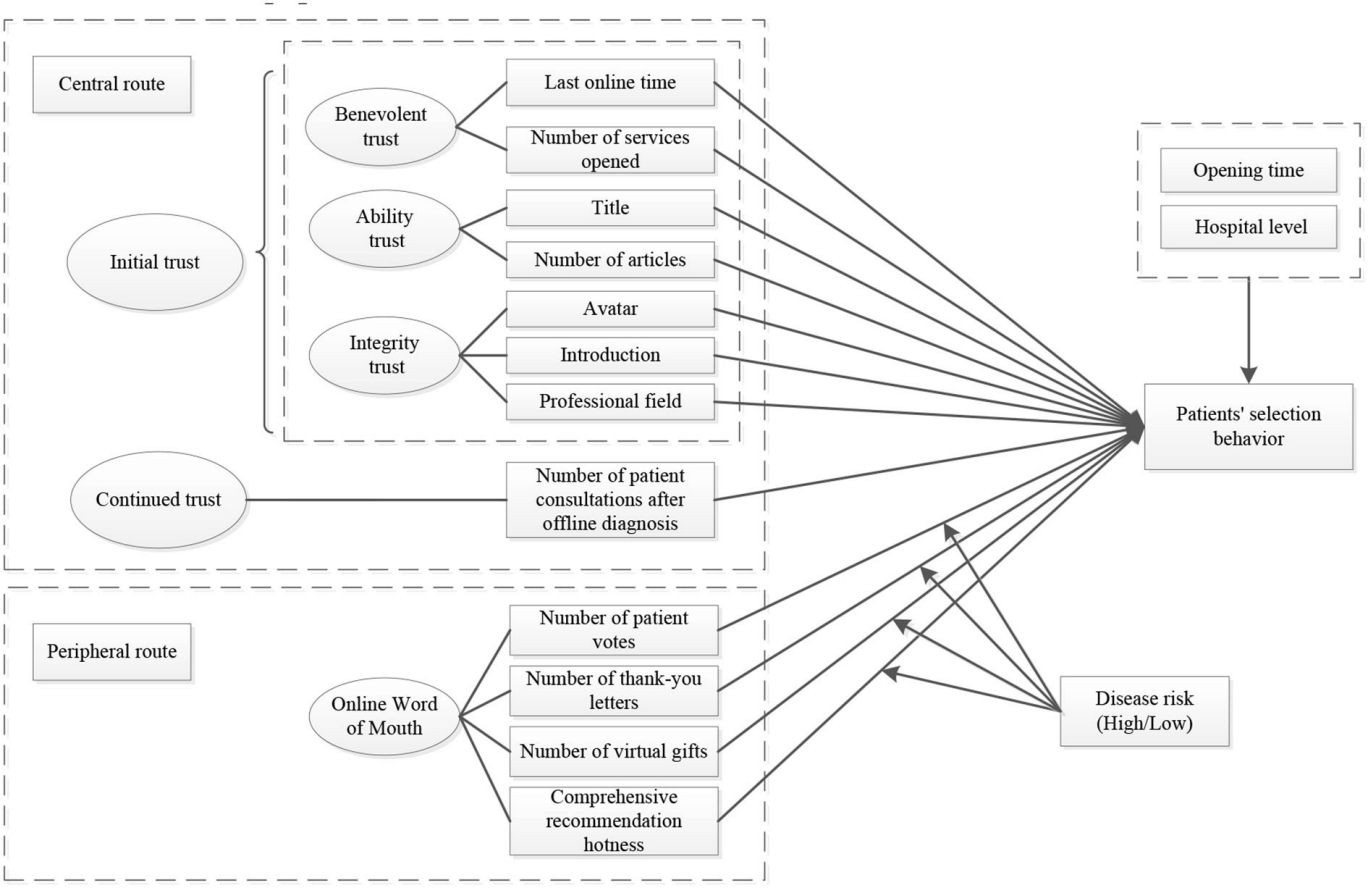
Conceptual model of patients' selection behavior in OHC.

Based on the above assumptions, the theoretical framework model shown in Figure 1 is constructed in this article.

## Research method

### Sample selection and data collection

Data were collected from the Good Doctor website (www.haodf.com) in China. Founded in 2006, Good Doctor is currently a fully functional and well-established physician–patient-based OHC. Good Doctor includes 890,000 doctors' information on the platform, and physicians have opened various online health services to meet the needs of different patients, whose main services include online consultation, team consultation, appointment booking, and private physician. The Good Doctor platform has been established for a long time, and has many physicians with high stickiness and a large patient user base. In addition, the personal information and OWOM information of physicians are open and transparent on their personal homepages, which makes it easy to obtain data and is suitable for the research object of this study.

**Figure 2:**
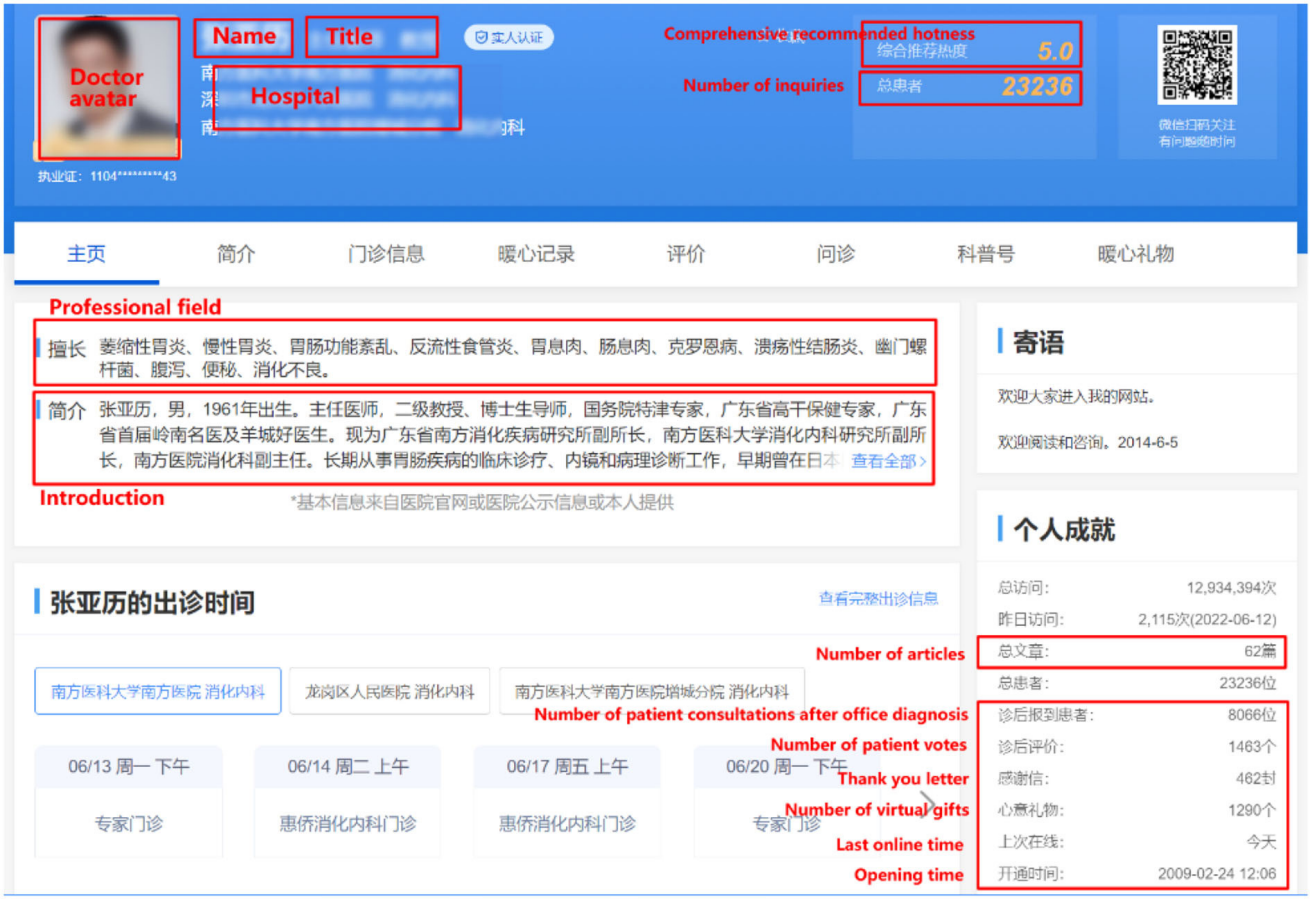
Good Doctor website physician information page.

The physician's personal homepage shows the following information in detail: the physician's avatar, title, hospital, professional field, introduction, service type, total consultations, total articles, patient votes, number of thank you letters, number of virtual gifts, and comprehensive recommendation hotness. A sample of Good Doctor's personal homepage is shown in Figure 2.

Disease risk refers to the mortality rate of a diagnosed disease, reflecting the severity of the disease. Based on the experience of previous studies (70, 73), we selected lung cancer as a high-risk disease and pneumonia as a low-risk disease. According to the Chinese Health and Health Statistics Yearbook 2021, lung cancer ranked first among malignant tumor deaths. In addition, common pneumonia, the most common respiratory disease, is an infectious disease with high curability and low mortality and is comparable with lung cancer as a respiratory disease. In particular, it is noted that pneumonia in this article is a common type of pneumonia that is different from COVID-19.

We used the Python crawler program to obtain the real physician data on the Good Doctor website, and crawled pneumonia and lung cancer treatment physicians according to the disease classification, crawling time for 1 December 2020, 1 January 2021–1 June 2021, the time interval of 1 month, a total of 13,955 physician data. After the data were coded for cleaning, removing invalid null data, completing missing data, and other cleaning data methods finally obtained 10,446 physician sample data, with an efficiency rate of 74.9%.

### Measure of variables

The specific variable descriptions are shown in Table 1. Physician titles are qualitative variables, which need to be quantified: chief physician = 4, associate chief physician = 3, attending physician = 2, resident physician = 1, and other = 0. The academic titles of physicians range from high to low: professor (researcher) = 4, associate professor (associate researcher) = 3, lecturer = 2, assistant professor = 1, and other = 0. And the two different levels of the system were normalized. As shown in equation (1).


(1) $$\begin{array}{l} TotalTitle=\frac{\left[DIG\left(MedTitle\right)+DIG\left(AcaTitle\right)\right]}{2} \end{array}$$

**Table 1:** Description of variables.

**Variable type**	**Variable name**	**Description**
Dependent variable	Selection	Number of patient inquiries
	Last_online	Physician's last website login time
	ServiceNum	The number of online health services opened by physicians, including online consultation, appointment booking, private physician, team consultation, 1 for opening a certain item, 0 for not opening, and the total sum is the number of opened services
	AcaTitle	Professor (Researcher) = 4, Associate Professor (Associate Researcher) = 3, Lecturer = 2, Assistant Professor = 1, Other = 0
	MedTitle	Chief Physician = 4, Associate Chief Physician = 3, Attending Physician = 2, Resident Physician = 1, others are 0
Independent variable	ArticleNum	Number of scientific articles by physicians
	Avatar	Whether there is an avatar, with avatar for 1, no avatar for 0
	Professional	professional field in distribution according to word count, 1 for the first 25%, 2 for 25–50%, 3 for 50–75%, and 4 for 75–100%
	Introduction	Introduction according to the word count distribution, the first 25% is 1, 25–50% is 2, 50–75% is 3, 75–100% is 4
	Reported patient	Number of patients reported after consultation with physicians
	Vote	Number of patient votes received by this physician
	Letter	Number of thank you letters received by this physician
	Gift	Number of virtual gifts received by physicians from patients
	Heat	Comprehensive recommended hotness
Adjustment variables	Risk	1 for high-risk lung cancer and 0 for low-risk pneumonia
Control variables	Open_time	Number of months between collection time and opening time
	Rank	Hospital level is divided into Grade A and other, Grade A is 1, other is 0

*TotalTitle* represents the title after normalization, and *DIG(MedTitle)* and *DIG(AcaTitle)* represent the quantification of medical and academic titles.

On the Good Doctor platform, physician avatars, professional fields, and introductions are uploaded or filled in at the discretion of the physician. For the processing of these online disclosures, the presence or absence of a physician's personal avatar is quantified numerically, with an avatar = 1 and no avatar = 0. The professional field is divided into profile ratings based on word count in a fixed order, with quartiles as the threshold, and 4–1 points, respectively, by word count, and personal introductions are quantified in the same way.

The control variables used in this study were the opening time of the physician's webpage and the hospital rank, where the hospital rank is based on the Chinese Hospital Rank Management Standard, which ranks hospitals into three levels: level 1 is the basic hospital, which usually provides medical services to the community, level 2 is the secondary hospital, and level 3 is the tertiary hospital. Level 3 hospitals employ more staff, have more beds, and are usually considered to provide a higher quality of service than the other two levels, so quantifying the hospital-level numbers, level 3 hospitals = 1 and the rest of the hospitals = 0. In our study model, these variables are used to control for the effect on patient selection.

The results of the descriptive statistics of the variables are shown in Table 2. The dependent and independent variables may not be normally distributed, so the dependent variable and the continuous-type independent variables that are positively skewed are transformed by taking the logarithm (ln(x+1)). The skewness of the variables was all controlled below 3 and could be placed in the regression model for subsequent analysis and testing.

**Table 2:** Descriptive statistics of variables.

**Variables**	***N***	**Minimum**	**Maximum**	**Mean value**	**Standard deviation**	**Skewness**
selection	10,446	0	36,900	1363.41	2666.342	5.166
open_time	10,446	0	158	90.47	42.875	−0.167
hospital	10,446	0	1	0.98	0.149	−6.412
last_online	10,446	0	4	2.57	1.841	−0.607
service	10,446	0	4	1.42	1.109	0.339
title	10,446	0.5	4.0	2.983	1.0867	−0.592
article	10,446	0	5,003	29.08	174.434	18.332
avatar	10,446	0	1	0.69	0.464	−0.801
professional	10,446	1	4	2.49	1.123	0.010
introduction	10,446	1	4	2.50	1.120	0.003
report	10,446	0	22,911	488.15	1228.069	8.369
vote	10,446	0	2,020	123.74	207.883	4.104
letter	10,446	0	1,109	59.70	108.419	4.355
gift	10,446	0	3,208	119.04	298.196	5.674
heat	10,446	2.9	5.0	3.817	0.4178	1.245

The correlation analysis of variables is shown in Table 3, and all independent variables were significantly correlated with the dependent variable, except for the control variable hospital grade, which was within the normal range of correlation.

**Table 3:** Correlation analysis.

	**1**	**2**	**3**	**4**	**5**	**6**	**7**	**8**	**9**	**10**	**11**	**12**	**13**	**14**	**15**	**16**
selection	1.000															
open_time	0.190^***^	1.000														
hospital	−0.008	0.001	1.000													
last_online	0.605^***^	−0.174^***^	0.004	1.000												
service	0.653^***^	−0.056^***^	−0.011	0.689^***^	1.000											
title	−0.084^***^	0.413^***^	−0.001	−0.343^***^	−0.238^***^	1.000										
article	0.631^***^	0.214^***^	−0.029^***^	0.406^***^	0.476^***^	−0.041^***^	1.000									
avatar	0.415^***^	−0.076^***^	−0.026^***^	0.435^***^	0.455^***^	−0.182^***^	0.360^***^	1.000								
professional	0.258^***^	0.037^***^	−0.061^***^	0.185^***^	0.281^***^	0.012	0.283^***^	0.153^***^	1.000							
introduction	0.059^***^	0.331^***^	0.027^***^	−0.145^***^	−0.030^***^	0.423^***^	0.134^***^	−0.035^***^	0.172^***^	1.000						
report	0.819^***^	−0.024^**^	0.000	0.633^***^	0.652^***^	−0.254^***^	0.519^***^	0.397^***^	0.249^***^	−0.063^***^	1.000					
vote	0.818^***^	0.161^***^	0.022^**^	0.537^***^	0.545^***^	−0.123^***^	0.512^***^	0.335^***^	0.252^***^	0.060^***^	0.833^***^	1.000				
gift	0.908^***^	0.135^***^	0.003	0.598^***^	0.615^***^	−0.160^***^	0.612^***^	0.400^***^	0.232^***^	0.021^**^	0.842^***^	0.876^***^	1.000			
heat	0.584^***^	0.008	0.026^***^	0.500^***^	0.465^***^	−0.082^***^	0.389^***^	0.268^***^	0.219^***^	0.037^***^	0.678^***^	0.733^***^	0.637^***^	1.000		
letter	0.825^***^	0.109^***^	0.026^***^	0.573^***^	0.581^***^	−0.173^***^	0.527^***^	0.354^***^	0.250^***^	0.028^***^	0.844^***^	0.978^***^	0.885^***^	0.729^***^	1.000	
risk	0.058^***^	0.064^***^	0.059^***^	0.104^***^	0.078^***^	0.062^***^	0.042^***^	0.068^***^	0.000	0.002	0.105^***^	0.193^***^	0.131^***^	0.236^***^	0.183^***^	1.000

*** p < 0.01,

** p < 0.05.

### Model construction

To test hypotheses H1a–H6d, we construct the following model. The mathematical model of the empirical study is presented in equation (2).


(2) $$\begin{array}{l} \ln\left(Selection_{it}+1\right)=\beta_{0}+\beta_{1}OpenTime_{it}+\beta_{2}HospitalRank_{i}\\ \;+\beta_{3}ServiceNum_{it}+\beta_{4}LastOnline_{it}\\ \;+\beta_{5}TotalTitle_{it}+\beta_{6}\ln\left(ArticleNum_{it}+1\right)\\ \;+\beta_{7}Avatar_{it}+\beta_{8}Professional_{it}\\ \;+\beta_{9}Introduction_{it}+\beta_{10}\ln(ReportedPatient_{it}+1)\\ \;+\beta_{11}\ln\left(VoteNum_{it}+1\right)+\beta_{12}\ln(LetterNum_{it}+1)\\ \;+\beta_{13}\ln\left(GiftNum_{it}+1\right)+\beta_{14}Heat_{it}+\beta_{15}Rsik_{i}\\ \;+\beta_{16}Risk_{i}\times \ln\left(VoteNum_{it}+1\right)+\beta_{17}Risk_{i}\times \\ \;\ln(LetterNum_{it}+1)+\beta_{18}Risk_{i}\times \ln(GiftNum_{it}+1)\\ \;\begin{array}{l} \;+\beta_{19}Risk_{i}\times Heat_{it}+\varepsilon_{it} \end{array} \end{array}$$

In the model, *Selection* represents the number of consultations by the physician, *OpenTime* represents the opening time of the physician's personal website, *HospitalRank* represents the hospital rank of the physician, *ServiceNum* represents the number of services opened by the physician, *LastOnline* represents the last time the physician was online, *TotalTitle* represents the physician's title, *ArticleNum* represents the number of articles published by the physician, *Avatar* represents whether the physician has a personal avatar, *Professional* represents the physician's professional field, *Introduction* represents the physician's introduction, *ReportedPatient* represents the number of patients reported by the physician after the initial consultation, *VoteNum* represents the number of patient votes the physician received, *LetterNum* represents the number of patients' thank you letters received by the physician, *GiftNum* represents the number of virtual gifts received by the physician, *Heat* represents the platform recommendation heat of the physician, *Riski* represents the risk size of the physician's specialization in the disease, β_0_*-*β_16_ represents the regression coefficient, ε represents the time perturbed error term, and *Risk* × *ln (VoteNum*+*1), Risk* × *ln (LetterNum*+*1), Risk* × *ln (GiftNum*+*1)*, and *Risk* × *Heat* test for the moderating effect of disease risk.

### Analysis of results

In this study, Stata 16.0 software was used to conduct multiple regression analysis on panel data to estimate the influencing factors of each variable in a hierarchical regression, as shown in Table 4. Model 1 contains only control variables, model 2 adds independent variables such as *ServiceNum*_*it*_ based on model 1, and model 3 adds four interaction terms on the basis of model 2, by the adjusted F-value is significant and *R*^2^ is significantly increased, indicating that the introduced explanatory variables have a strong explanatory effect on the explained variables and the model fit is better.

**Table 4:** Regression results.

**Variables**	**Model 1**	**Model 2**	**Model 3**
open_time	0.0112^***^	0.00232^***^	0.00236^***^
	(4.06)	(4.88)	(4.81)
hospital	0.0294^**^	0.0117	0.0125
	(2.15)	(0.27)	(0.29)
last_online	—	0.00135	0.00119
		(1.01)	(0.90)
service	—	0.0295^***^	0.0279^***^
		(5.86)	(5.59)
title	—	−0.0253^***^	−0.0225^**^
		(−2.62)	(−2.35)
ln (article+1)	—	0.0842^***^	0.0863^***^
		(8.64)	(8.90)
avatar	—	0.0860^***^	0.0844^***^
		(6.68)	(6.62)
introduction	—	−0.00956^***^	−0.00819^**^
		(−2.68)	(−2.31)
professional	—	0.00443	0.00529
		(0.54)	(0.65)
ln (report+1)	—	0.178^***^	0.172^***^
		(24.95)	(24.22)
ln (vote+1)	—	0.288^***^	0.137^***^
		(13.75)	(4.47)
ln (letter+1)	—	0.304^***^	0.0260
		(11.72)	(0.53)
ln (gift+1)	—	0.249^***^	0.159^***^
		(24.49)	(10.81)
heat	—	0.0977^***^	0.125^***^
		(7.76)	(7.67)
risk × ln (vote+1)	—	—	0.235^***^
			(5.86)
risk × ln (letter+1)	—	—	0.376^***^
			(6.31)
risk × ln (gift+1)	—	—	0.155^***^
			(7.78)
risk × heat	—	—	0.0764^***^
			(2.93)
Constant	4.638^***^	3.251^***^	3.208^***^
	(17.78)	(41.48)	(39.00)
*N*	10,446	10,446	10,446
*R* ^2^	0.030	0.384	0.395
*F*	17.80^***^	388.31^***^	316.10^***^

t-values in parentheses;

*** p < 0.01,

** p < 0.05.

From the regression results of model 2, it can be seen that there is no significant relationship between patients' selection behavior and physicians' last online time (β = 0.0013, sig = 0.311>0.1), and hypothesis H1a is not supported, indicating that patients do not value physicians' last online time in selecting physicians on medical platforms. The number of physicians opening services representing benevolent trust significantly and positively influenced patients' selection behavior (β = 0.0295, sig = 0.000<0.01), and hypothesis H1b was supported. It indicates that in the face of patients with different needs, physicians with a high number of open services can meet different needs, and patients are more inclined to select physicians with a rich number of services. The physician's title, which represents trust in competence, negatively influenced patients' selection behavior (β = −0.0253, sig = 0.009<0.01), and hypothesis H2a was not supported. Higher medical and academic titles of physicians instead lead to fewer patients consulting with that physician, possibly because physicians with higher titles have busier offline work, such as managing a hospital or teaching at a university. The number of articles representing ability trust significantly and positively influenced patients' selection behavior (β = 0.0842, sig = 0.000<0.01), and hypothesis H2b was supported. That is, the higher the number of articles published by physicians on the platform, the stronger the ability to trust that patients can perceive. The physician avatar representing integrity trust significantly and positively influenced patients' selection behavior (β = 0.0860, sig = 0.000<0.01), and hypothesis H3a was supported. Patients feel more authentic when physicians put their personal avatars on their personal homepages, and the sense of unknown and fear during a consultation is diminished. The personal introduction written by the physician has a significant negative effect on patients' selection behavior (β = −0.0096, sig = 0.007<0.01), and hypothesis H3b is not supported. When the content of a physician's personal introduction is excessive and complicated, patients may feel that the physician's expression is tedious and makes it impossible to find the core content, instead, a short and concise introduction is more likely to attract patients' attention. There was no significant effect of the physician's description of professional expertise on patients' selection behavior (β = 0.0045, sig = 0.591>0.1), and hypothesis H3c was not supported. When a physician's professional field is described too broadly, patients may feel that the physician is studying too many directions and is unable to focus on a particular medical technique, being general rather than specialized. The number of patient consultations after office diagnosis significantly and positively influenced patients' selection behavior (β = 0.178, sig = 0.000<0.01), and hypothesis H4 was supported. Patients' recognition that many patients similar to themselves have consulted the physician strengthens the intensity of their internal trust in the physician and increases the likelihood of consulting services.

In response to the effect of OWOM on patient selection behavior, the number of patient votes for physicians significantly and positively influenced patient selection behavior (β = 0.288, sig = 0.000<0.01), and hypothesis H5a was supported. A higher number of votes from physicians evidenced more patient recommendations, thus promoting patient selection. The number of thank-you letters from physicians significantly and positively influenced patient selection behavior (β = 0.304, sig = 0.000<0.01), and hypothesis H5b was supported. The number of heartfelt gifts from physicians significantly and positively influenced patient selection behavior (β = 0.249, sig = 0.000<0.01), and hypothesis H5c was supported. Heartfelt gifts are required to be purchased on the Good Doctor platform. Compared with the number of patient votes and thank-you letters, the number of gifts highlights the level of physician service at the level of price and reflects patients' emotional inclination toward physicians. The platform's comprehensive recommendation hotness also significantly positively affected patients' selection behavior (β = 0.0977, sig = 0.000<0.01), and hypothesis H5d was supported. The comprehensive recommendation hotness is the rating of physicians by the Good Doctor platform based on their past performance, which reflects the superiority of physicians from the validity level and has a certain “celebrity doctor effect” to attract more patients to select. From the regression coefficients, we can see that the number of physicians' OWOM is more influential than the platform's comprehensive recommendation hotness, probably because the number of physicians' OWOM is generated from patients who have previously consulted with them, which is more convincing to potential patients.

The regression results from model 3 showed that for the influences on the peripheral route, disease risk significantly and positively moderated the number of patient votes (β = 0.235, sig = 0.000<0.01) and virtual gifts (β = 0.155, sig = 0.000<0.01) as well as the comprehension recommendation hotness (β = 0.0764, sig = 0.003<0.01) on patient selection behavior, hypotheses H6a, H6c, and H6d were supported. When patients have high-risk diseases, due to the complexity of the disease, most patients are unable to judge through their existing knowledge and experience, and therefore may rely more on OWOM information from third parties. OWOM information to a certain extent also reflects the physician's professional and technical ability and service communication ability. Therefore, for high-risk diseases, patients will consider their physicians' personal information more thoroughly, and the moderating effect of OWOM cannot be ignored. The moderating effect of disease risk on the number of thank-you letters and patients' selection behavior was not significant (β = 0.026, sig>0.1), and hypothesis H6b was not supported. The possible reason is that patients suffering from low-risk diseases do not have a certain level of appreciation for physician treatment, reducing the effect on patients' selection behavior.

The moderating effect of disease risk on OWOM information of physicians in the peripheral route was further explored by subgroup regression. The results of the subgroup regressions are shown in Table 5. As shown by the comparison of the subgroup regression results, for high-risk diseases, physician title and professional field had no significant effect on patient selection behavior, while for low-risk diseases, both physician title (β = −0.0376, sig = 0.009<0.01) and professional field (β = −0.0418, sig = 0.01<0.05) had a significant negative effect on patients' selection behavior. The possible reason is that patients with low-risk diseases have some knowledge about the disease and do not pay much attention to the physician's title and professional field, but focus on quick and easy access to medical services. There was no significant effect of physician avatar on patients' selection behavior in high-risk diseases, while there was a significant positive effect on patients' selection behavior in low-risk diseases (β = 0.0687, sig = 0.002<0.01). This suggests that patients with low-risk diseases care more about the physician who displays their avatar, which makes them perceive closeness and increases the likelihood of selecting a physician. In terms of influencing factors on the peripheral route, for high-risk diseases, the number of thank-you letters significantly and positively influenced patients' selection behavior (β = 0.437, sig = 0.000<0.01); for low-risk diseases, the number of thank-you letters did not have a significant effect on patients' selection behavior. Compared to patients with low-risk diseases, patients with high-risk diseases had a sense of “life after surviving a disaster” after treatment and were more likely to express their gratitude to physicians in the form of thank-you letters than patients with low-risk diseases. Also, the results of the group regression are consistent with the results of the previous model, indicating that the model is robust.

**Table 5:** Regression results of subgroups.

**Variables**	**High-risk (lung cancer)**	**Low-risk (pneumonia)**
open_time	0.00389^***^	0.00114
	(7.37)	(1.36)
Hospital	0.0121	0.0110
	(0.22)	(0.18)
last_online	0.00134	0.000171
	(0.89)	(0.08)
service	0.0207^***^	0.0245^**^
	(4.16)	(2.70)
title	0.00674	−0.0376^***^
	(0.58)	(−2.60)
ln(article+1)	0.0423^***^	0.136^***^
	(4.13)	(8.14)
avatar	0.00513	0.0687^***^
	(0.38)	(3.11)
introduction	−0.00979^***^	−0.00651
	(−2.77)	(−1.01)
professional	0.00853	−0.0418^**^
	(1.08)	(−2.57)
ln (report+1)	0.118^***^	0.148^***^
	(18.55)	(8.29)
ln (vote+1)	0.381^***^	0.159^***^
	(17.50)	(4.20)
ln (letter+1)	0.437^***^	−0.0494
	(16.91)	(−0.86)
ln (gift+1)	0.350^***^	0.170^***^
	(31.95)	(9.54)
heat	0.0766^***^	0.132^***^
	(4.88)	(6.70)
Constant	3.314^***^	3.487^***^
	(34.29)	(26.51)
*N*	5,616	4,598
*R* ^2^	0.579	0.238
*F*	460.07^***^	85.60^***^

t-values in parentheses;

*** p < 0.01,

** p < 0.05.

## Discussion and implications

### Conclusion

Due to information asymmetry between the two parties, the current state of physician–patient distrust exists in online healthcare services (31, 74). This article uses ELM as the structure, the central route is based on trust source theory and the peripheral route is based on OWOM theory. Data collected from Good Doctor with quantitative analysis methods are used to formulate the appropriate research models using variables for the context. We construct a multiple linear regression model and empirically test the proposed research hypotheses. The results are shown in Table 6, and most of the hypotheses are supported.

**Table 6:** Summary of results.

**Hypothesis description**	**Result**
**H1a:** The more recent the physician was last online, the more patients selected that physician	Not Supported
**H1b:** The number of services opened by the physician positively influences the patient's selection behavior.	Supported
**H2a:** Physician's title positively influences patients' selection behavior.	Not Supported
**H2b:** The number of physician articles positively influences patients' selection behavior.	Supported
**H3a:** Physicians use of avatars positively influences patients' selection behavior.	Supported
**H3b:** Physician's introduction positively influences patients' selection behavior.	Not Supported
**H3c:** Physician's professional field positively influences patients' selection behavior.	Not Supported
**H4:** The number of patients consultations after physician's offline diagnosis positively influences patients' selection behavior.	Supported
**H5a:** The number of patients' votes for physicians positively influences patients' selection behavior.	Supported
**H5b:** The number of thank-you letters of physicians positively influences patients' selection behavior.	Supported
**H5c:** The number of virtual gifts of physicians positively influences patients' selection behavior.	Supported
**H5d:** The comprehensive recommendation hotness of physicians positively influences patients' selection behavior.	Supported
**H6a:** Disease risk significantly moderates the relationship between the number of patients votes and patients' selection behavior.	Supported
**H6b:** Disease risk significantly moderates the relationship between the number of thank-you letters and patients' selection behavior.	Not Supported
**H6c:** Disease risk significantly moderates the relationship between the number of virtual gifts and patients' selection behavior.	Supported
**H6d:** Disease risk significantly moderates the relationship between comprehensive recommendation hotness and patients' selection behavior.	Supported

The main findings of the study: first, the number of opened services, articles, and avatars in the central route all had a significant positive effect on patients' selection behavior. The title and personal introduction had a significant negative effect on patients' selection behavior. According to the personal information of physicians, the more types of services opened by physicians, the more articles published, and the use of personal avatars promoted patients' initial trust in physicians and thus their selection of them for medical consultation services. Higher titles and more complex personal introductions, on the contrary, reduce potential patients' initial trust in the physician and constrain patients' selection. The number of patients reported after the consultation represents patients who continue to trust the physician. The higher the number of patients reported after the consultation, the more patients approve of the medical services provided by the physician, increasing the occurrence of potential patients' selection behavior. Second, both patient-generated OWOM information and platform scoring significantly contribute to patient selection behavior, and patient-generated OWOM information has a greater impact on patient decision-making. Finally, disease risk significantly moderates the relationship between OWOM information and patients' selection behavior. Patients with high-risk diseases have less knowledge about the disease and pay more attention to the experience sharing given by others to assess the physician's medical skill, while patients with low-risk diseases have more knowledge about the disease and pay more attention to the physician's personal information when selecting a physician.

### Theoretical contribution

This article makes several theoretical contributions to the literature.

First, we propose a relatively comprehensive model of the factors influencing patient selection behavior based on ELM. Previous studies on the patient selection are mostly based on trust theory and social exchange theory to explore the influence of peripheral routes such as OWOM on patient selection (11, 19, 51). This article considers the trusted source of physicians' personal information as a central route factor, as well as OWOM marginal routes. It enriches the theoretical research in the field of patients' selection behavior.

Second, previous studies on physician–patient trust have focused on patient-generated information (30, 75) with the few studies on physician information only discussing the relationship between the three elements of trust and physician–patient interactions (11). This article divides physician–patient trust into initial and continued trust according to the development stage of the physician–patient relationship (61). Additionally, it explores the influence of the three elements of initial trust source and the number of patients' consultations after diagnosis on the physician–patient relationship in continued trust. The study enriches the research in the field of physician–patient trust.

Third, this article considers disease risk as a moderating variable between OWOM information of physicians and patients' selection behavior. Previous research on the impact of OWOM on patient selection behavior has focused on the moderating effect of demographic information such as gender, age, and education level (11, 21). This study explores the moderating role of disease risk by examining the difference in patients' selection of physicians under two different disease risks. It expands the application of OWOM in OHC.

### Managerial implications

The findings of this study provide several managerial insights.

First, it can provide patients with a basis for selecting quality physicians and improve their trust in physicians when making decisions. Patients should focus on the personal information data of the physician such as avatar, the number of services opened, the number of articles, and the number of patients reported after the consultation. These can be used to identify quality physicians with proper medical skills and ethics. At the same time, we should pay attention to the physician's online reputation and the evaluation of similar patients is an important basis to help patients make decisions.

Second, for physician services, it encourages physicians to improve the quality of online services through increased participation in the platform, requiring them to improve their homepages as well as maintain a good relationship with patients. Physicians should pay attention to the improvement and management of personal information, choose an affable avatar display, and a more concise resume; within the scope of their ability, they should carry out diversified types of services, while improving their level of professional competence and focusing on personal OWOM management.

Third, for the platform construction, it helps to design and improve the platform mechanism to provide more sound services for physician–patient communication. Enhancing the disclosure of physicians' personal information, increasing the display of objective data of physicians' online services, and improving the evaluation indexes of the platform will help patients enhance their trust in physicians and help them make decisions.

### Limitations and future research

Due to the limitations of the research method and subjects, there are areas for improvement in this study. First, the study data were only from two diseases on the Good Doctor platform, which is somewhat one-sided, and the study results lacked wide generalizability. In future, we may consider selecting physician data from multiple platforms for integrated analysis or selecting multiple high and low-risk disease groups for exploration. Second, the study only considered the objective data of physicians, and since online medical services need to be purchased to obtain, there is a lack of reference to the content of physician–patient interaction, and the interactive content can be incorporated into the model for impact factor analysis in future.

## Data availability statement

The raw data supporting the conclusions of this article will be made available by the authors, without undue reservation.

## Author contributions

Study conception and design: MQ. Data collection: WZ, CY, SL, and SQ. Data analysis and drafting of the article: CY and WZ. Critical revision of the article and accepts all responsibility for the work and/or the conduct of the study, had access to the data, and controlled the decision to publish: WZ. All authors read and approved the final manuscript.

## Funding

This research was supported by the National Natural Science Foundation of China (Grant No. 71762018), Jiangxi University Humanities and Social Science Research Project (Grant No. GL20132), and Jiangxi Province Graduate Education Reform Research Project Key Project (Grant No. JXYJG-2020-041).

## Conflict of interest

The authors declare that the research was conducted in the absence of any commercial or financial relationships that could be construed as a potential conflict of interest.

## Publisher's note

All claims expressed in this article are solely those of the authors and do not necessarily represent those of their affiliated organizations, or those of the publisher, the editors and the reviewers. Any product that may be evaluated in this article, or claim that may be made by its manufacturer, is not guaranteed or endorsed by the publisher.
